# Disparities in Cancer Stage Outcomes by Catchment Areas for a Comprehensive Cancer Center

**DOI:** 10.1001/jamanetworkopen.2024.9474

**Published:** 2024-05-02

**Authors:** Michael R. Desjardins, Norma F. Kanarek, William G. Nelson, Jamie Bachman, Frank C. Curriero

**Affiliations:** 1 Department of Epidemiology and Spatial Science for Public Health Center, Johns Hopkins Bloomberg School of Public Health, Baltimore, Maryland; 2 Department of Environmental Health Sciences, Johns Hopkins Bloomberg School of Public Health, Baltimore, Maryland; 3 Sidney Kimmel Comprehensive Cancer Center, Johns Hopkins School of Medicine, Baltimore, Maryland; 4 Department of Oncology, Johns Hopkins School of Medicine, Baltimore, Maryland

## Abstract

**Importance:**

The National Cancer Institute comprehensive cancer centers (CCCs) lack spatial and temporal evaluation of their self-designated catchment areas.

**Objective:**

To identify disparities in cancer stage at diagnosis within and outside a CCC’s catchment area across a 10-year period using spatial and statistical analyses.

**Design, Setting, and Participants:**

This cross-sectional, population-based study conducted between 2010 and 2019 utilized cancer registry data for the Johns Hopkins Sidney Kimmel CCC (SKCCC). Eligible participants included patients with cancer in the contiguous US who received treatment for cancer, a diagnosis of cancer, or both at SKCCC. Patients were geocoded to zip code tabulation areas (ZCTAs). Individual-level variables included sociodemographic characteristics, smoking and alcohol use, treatment type, cancer site, and insurance type. Data analysis was performed between March and July 2023.

**Exposures:**

Distance between SKCCC and ZCTAs were computed to generate a catchment area of the closest 75% of patients and outer zones in 5% increments for comparison.

**Main Outcomes and Measures:**

The primary outcome was cancer stage at diagnosis, defined as early-stage, late-stage, or unknown stage. Multinomial logistic regression was used to determine associations of catchment area with stage at diagnosis.

**Results:**

This study had a total of 94 007 participants (46 009 male [48.94%] and 47 998 female [51.06%]; 30 195 aged 22-45 years [32.12%]; 4209 Asian [4.48%]; 2408 Hispanic [2.56%]; 16 004 non-Hispanic Black [17.02%]; 69 052 non-Hispanic White [73.45%]; and 2334 with other or unknown race or ethnicity [2.48%]), including 47 245 patients (50.26%) who received a diagnosis of early-stage cancer, 19 491 (20.73%) who received a diagnosis of late-stage cancer , and 27 271 (29.01%) with unknown stage. Living outside the main catchment area was associated with higher odds of late-stage cancers for those who received only a diagnosis (odds ratio [OR], 1.50; 95% CI, 1.10-2.05) or only treatment (OR, 1.44; 95% CI, 1.28-1.61) at SKCCC. Non-Hispanic Black patients (OR, 1.16; 95% CI, 1.10-1.23) and those with Medicaid (OR, 1.65; 95% CI, 1.46-1.86) and no insurance at time of treatment (OR, 2.12; 95% CI, 1.79-2.51) also had higher odds of receiving a late-stage cancer diagnosis.

**Conclusions and Relevance:**

In this cross-sectional study of CCC data from 2010 to 2019, patients residing outside the main catchment area, non-Hispanic Black patients, and patients with Medicaid or no insurance had higher odds of late-stage diagnoses. These findings suggest that disadvantaged populations and those living outside of the main catchment area of a CCC may face barriers to screening and treatment. Care-sharing agreements among CCCs could address these issues.

## Key Points

**Question:**

Are there disparities in cancer staging within and outside a comprehensive cancer center’s catchment area?

**Findings:**

In this cross-sectional study of 94 007 patients at the Sidney Kimmel Comprehensive Care Center, statistically significant disparities in cancer staging were identified, including higher odds of late-stage cancers for non-Hispanic Black patients, those with Medicaid and no insurance, and patients residing outside the main catchment that either only received treatment or only received a diagnosis at the center.

**Meaning:**

These findings suggest that disadvantaged populations and those living outside of a comprehensive cancer center’s main catchment area may face barriers to screening and treatment, resulting in higher odds of receiving a diagnosis of late-stage cancer.

## Teaser

This cross-sectional study analyzes disparities in cancer stage at diagnosis within and outside the catchment area of a comprehensive cancer center across a 10-year period.

## Introduction

The National Cancer Institute (NCI) has designated 72 cancer centers since the creation of the National Cancer Act of 1971^1^ with the goal of identifying centers that focus on transdisciplinary, cutting-edge research to prevent, diagnose, and treat cancer.^2^ Each cancer center serves a particular catchment area (CA) that is self-defined and contains most of their patients; these centers examine the cancer burden, risk factors, incidence, morbidity, mortality, and inequities.^3^ Among the 72 NCI-designated cancer centers, 54 are classified as comprehensive cancer centers (CCCs), which are especially recognized for the wide variety of resources, leadership, research across numerous disciplines, and outreach to serve underrepresented populations.^4^ Studies have shown that patients not receiving their first treatment at a CCC experience worse cancer outcomes, especially survivability.^5,6^ However, barriers to care, such as the association of geographic distance with increased odds of late-stage cancers and decreased survival^7,8^ and racial and ethnic and insurance disparities,^9,10,11,12,13,14^ are still a challenge.

It is critical to appropriately define CAs to optimize the evaluation of patient characteristics and outcomes over time to facilitate improved outreach, prevention, treatment, and survival. Because cancer centers self-define their own CAs, there is no objective approach to formalizing boundaries that also may change over time due to dynamics in patient accessibility and utilization. Some examples of self-defined CAs include using a case density approach to identify counties that have a high proportion of a center’s patients with cancer compared with all patients with cancer,^15^ using SaTScan cluster detection software to identify counties that have a higher than expected ratio of center cancer cases compared with all cancer deaths,^16^ using Bayesian hierarchical models to identify counties with higher than expected probabilities of patients diagnosed at a center,^17^ selecting counties that contributed 75% or more of the market share of cancer cases for a center,^18^ and identifying counties that participated in a multiinstitution cancer coalition program.^19^ These approaches differ from floating CA approaches in the spatial accessibility literature,^20,21,22^ which are mainly concerned with potential access to health care facilities rather than incorporating patient utilization data.

However, the aforementioned approaches are static and do not capture changes in the patient population over time; may result in disjoint boundaries; do not account for travel distance to seek screening, diagnosis, and treatment; and do not capture the dynamics of smaller administrative boundaries (eg, zip code tabulation areas [ZCTAs]) to capture within-county variations. As such, we have developed a simplified approach to define and evaluate CAs of a CCC across 2 time periods that considers (1) geographic distribution of cases, (2) travel distance, (3) smaller geographic units than counties, and (4) temporal changes in CA boundaries by examining patterns across a decade of cancer registry data. The main CA was defined as the closest 75% of patients (in miles) at time of diagnosis. Other geographic zones outside of the main CA were computed (eg, >75%-95% of the closest patients) to identify potential staging disparities of patients residing within and outside of the main CA. Our subsequent modeling approach considered numerous individual-level factors associated with early and late-stage cancers at time of diagnosis (eg, insurance type, and race and ethnicity). Our objective was to identify if residing outside of the main CA was associated with higher odds of a late-stage diagnosis, especially for those who solely received a diagnosis or solely received treatment at the CCC. We hypothesized that patients residing outside of the main CA that were both diagnosed and treated at our CCC would have lower odds of a late-stage diagnosis.

## Methods

This cross-sectional study was approved by the Johns Hopkins Bloomberg School of Public Health institutional review board. We followed the Strengthening the Reporting of Observational Studies in Epidemiology (STROBE) reporting guideline to ensure the quality of data reported in this study.

### Study Design and Patients

In this study, we examined the patient population, geographic distribution, and cancer outcomes and risk factors of patients at the Johns Hopkins Sidney Kimmel CCC (SKCCC) across a 10-year period (2010-2019) using The Johns Hopkins Hospital cancer registry data in the contiguous US. Founded in 1973, SKCCC was one of the first cancer centers designated by the NCI and currently treats over 2 dozen types of cancer.^23^ There are 5 main hospital campuses: (1) The Johns Hopkins Hospital in Baltimore, Maryland, (2) Bayview Medical Center in Baltimore, Maryland, (3) Howard County Hospital in Columbia, Maryland, (4) Suburban Hospital in Bethesda, Maryland, and (5) Sibley Memorial Hospital in Washington, D.C.

The Johns Hopkins Hospitals cancer registry data include sex at birth (male or female), age at diagnosis, race and Hispanic ethnicity, insurance type, cancer type, treatment type (including surgery, radiation, immunotherapy, hormone therapy, and chemotherapy), class of case (diagnosis and treatment at SKCCC, diagnosis only at SKCCC, treatment only at SKCCC, no treatment, and nonanalytical), and cancer staging. Chronic lymphocytic leukemia was staged using the Rai system, while all other leukemias were grouped under unknown stage. Race and ethnicity were reported via electronic medical records in the cancer registry. Race and ethnicity categories included Asian, Hispanic, Native American, Non-Hispanic Black, Non-Hispanic White, other race and ethnicity, and unknown. The category of other race did not contain any further information; therefore, we did not have the necessary metadata to determine which subcategories of race and ethnicity were included as other. Race and ethnicity were included in the study to to account for potential disparate outcomes in stage at diagnosis, treatment, and accessibility to SKCCC. The no treatment category was defined as the patient recorded as not receiving first course of treatment, and diagnosis only at SKCCC refers to the receipt of first course of treatment at a non-SKCCC facility (ie, elsewhere).

#### CA Definition

The zip code at diagnosis was converted to a ZCTA using the Uniform Data System cross walk file^24^ because zip codes do not have physical boundaries, whereas ZCTAs approximate the boundaries for analysis. We then computed the road-network distance between the population-weighted centroid of the ZCTA where each patient resided and the SKCCC geopraphic mean center to capture more realistic travel distances. As a result, each patient was assigned a travel distance to SKCCC in miles. We then computed the main SKCCC CA, defined as the closest 75% of patients by road-network distance, then outer zones (from >75%-95%) in 5% increments. Our CA approach aligns with initial suggestions by the NCI, which indicated that approximately 75% of patients should belong in the specified CA (although this is not enforced in practice).^25^ Our 75% CA was designated as the main CA and reference group, while patients outside of the 95% zone were assigned greater than 95% (ie, >95%) for subsequent modeling. CAs and outer zones were computed for each 5-year cross-section (2010-2014 and 2015-2019), depending on which year the patient received a diagnosis of cancer at SKCCC. Therefore, the main 75% CA and outer zones can vary by temporal cross-section to consider the utilization of SKCCC over time.

Our dependent variable was cancer stage, categorized as early (stages 0-II), late (stages III-IV), and unknown due to the large proportion of cases not having a stage at diagnosis. ZCTA of residence at diagnosis was also included and used for subsequent spatial analysis of CA definition and evaluation. To evaluate changes over time, we grouped the 10 years of data for year at diagnosis into two, 5-year cross-sections: 2010-2014 and 2015-2019.

### Statistical Analysis

Data analysis was performed between March and July 2023. We first computed descriptive statistics for each factor and our main outcome (ie, early, late, and unknown stage), which were further stratified into the previously defined CA and outer zones, time period at diagnosis, race and ethnicity by staging, and race and ethnicity by insurance type. For modeling purposes, we further grouped the categories by zone (75% CA, >75%-95%, and >95%). Our main analytical approach was multinomial logistic regression to identify if residing outside of the main CA was associated with higher odds of a late-stage diagnosis. We computed a variety of models: multivariable with interaction terms for (1) 2010 to 2014 and (2) 2015 to 2019, and (3) a full multivariable with interaction terms model where 2015 to 2019 was considered as a dummy variable. Odds ratios (ORs) and 95% CIs are reported. Interaction terms (75% CA and outer zones by class of case and CA and zone by race and ethnicity) were also examined. All data processing and regression modeling were conducted in R statistical software version 4.3.1 in RStudio version 2022.07.2 (R Project for Statistical Computing); geocoding, ZCTA aggregation, road network distance calculation, CA and patient zone generation, and resulting maps were prepared in ArcGIS desktop version 10.7.1 (Esri).

## Results

This study included a total of 94 007 patients (46 009 male [48.94%] and 47 998 female [51.06%]; 30 195 aged 22-45 years [32.12%]; 4209 Asian [4.48%]; 2408 Hispanic [2.56%]; 16 004 non-Hispanic Black [17.02%]; 69 052 non-Hispanic White [73.45%]; and 2334 with other or unknown race or ethnicity [2.48%]), including 47 245 patients (50.26%) who received a diagnosis of early-stage cancer, 19 491 patients (20.73%) who received a diagnosis of late-stage cancer, and 27 271 patients (29.01%) with unknown stage at diagnosis. The Figure (A) visualizes the ZCTAs belonging to the 75% CA and greater than 75% to 95% zones (in 5% increments) for SKCCC for patients who received a diagnosis between 2010-2014. Of the 296 ZTCAs in the 75% CA, 216 were in central and northern Maryland (72.97%), 58 were in northern Virginia (19.59%), and 22 were in Washington, D.C. (7.43%). The 75% to 80% zone included an additional 61 ZCTAs, with 40 in Maryland (65.57%; including 1 in the Eastern Shore), 1 in southern Pennsylvania (1.64%), and 20 in northern Virginia (32.79%). The 80% to 85% zone captured an additional 108 ZCTAs with 53 across Maryland (49.07%), 32 in southern Pennsylvania (29.63%), 21 in northern Virginia (19.44%), and 2 in West Virginia (1.8%). The 85% to 90% zone included an additional 175 ZCTAs, with 25 in Delaware (14.28%), 50 in Maryland (28.57%; mainly on the Eastern Shore), 57 in southern Pennsylvania (32.57%), 29 in Virginia (16.57%), and 68 in West Virginia (8.01%). The 90% to 95% zone captured an additional 260 ZCTAs, with 27 in Delaware (10.38%), 28 in Maryland (10.77%), 19 in southern New Jersey (7.31%), 141 in Pennsylvania (54.23%), 35 in Virginia (13.46%), and 10 in West Virginia (3.85%). Finally, 1339 ZCTAs fell outside the 95% zone across the US.

**Figure.**
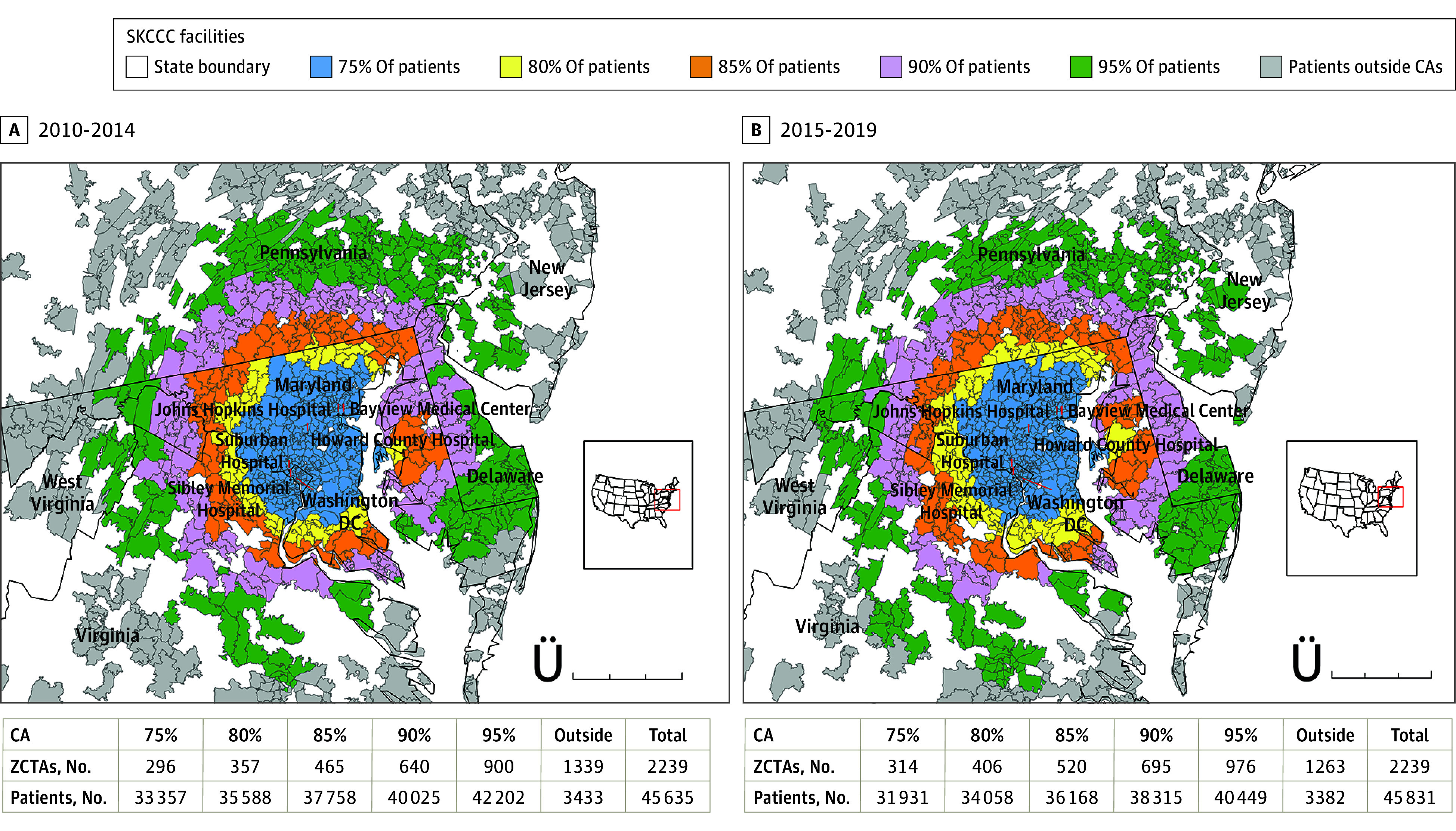
Main Catchment Area and Outsize Zones for the Sidney Kimmel Comprehensive Care Center (SKCCC) The figure shows the main catchment area (CA) for patients diagnosed from 2010 to 2014 (A) and 2015 to 2019 (B). Zip code tabulation areas (ZCTAs) were computed to generate a catchment area of the closest 75% of patients, and outer zones in 5% increments for comparison.

The Figure (B) visualizes the ZCTAs belonging to the 75% CA and outer zones for patients who received a diagnosis between 2015 and 2019. The 75% CA included 314 ZCTAs, with 22 in Washington, D.C. (7.01%), 230 in Maryland (73.25%), and 62 in northern Virginia (19.75%). The 75% to 80% zone included an additional 92 ZCTAs, with 51 in Maryland (55.43%), 13 in southern Pennsylvania (14.13%), and 28 in northern Virginia (30.43%). The 80% to 85% zone captured an additional 114 ZCTAs, with 48 in Maryland (42.1%), 38 in southern Pennsylvania (33.33%), 21 in northern Virginia (18.42%), and 7 in West Virginia (6.14%). The 85% to 90% zone included an additional 175 ZCTAs, with 37 in Delaware (21.14%), 38 in Maryland (21.71%), 1 in New Jersey (0.57%), 67 in southern Pennsylvania (38.28%), 23 in northern Virginia (13.14%), and 9 in West Virginia (5.14%). The 90% to 95% zone captured an additional 281 ZCTAs, with 15 in Delaware (5.34%), 32 in Maryland (11.39%), 25 in New Jersey (8.90%), 141 in Pennsylvania (50.18%), 53 in VA (18.86%), and 15 in West Virginia (5.33%). Finally, 1263 ZCTAs fell outside the 95% zone across the US.

Table 1 provides descriptive statistics for SKCCC patient characteristics stratified by the 75% CA and outer zone groups used for subsequent modeling. See eTable 1 in Supplement 1 for more detailed descriptive statistics with all variables examined stratified by time cross-section (ie, 2010-2014 and 2015-2019). Of the 94 007 patients at SKCCC, 47 002 (49.99%) were seen between 2010 and 2014 and 47 005 (50.01%) were seen between 2015 and 2019. The numbers of patients with early- and late-stage cancers seen at SKCCC increased between the 2 cross-sections, with 10.76% more overall between 2015 and 2019 (31 664 of 47 002 patients in 2010-2014 to 35 071 of 47 005 patients in 2015-2019). The number of patients with an unknown cancer stage decreased by 22.20% compared with 2010-2014 (15 338 of 47 002 patients in 2010-2014 vs. 11 933 out of 47 005 patients in 2015-2019). The majority of patients outside of the 95% zone were diagnosed with an earlier stage cancer across both cross-sections. Most patients were older than 45 years of age and non-Hispanic White, with a 15.52% increase in racial and ethnic minority groups between 2015 and 2019 (11 579 patients in 2010-2014 vs. 13 376 patients in 2015-2019). We reported 17 types of cancer, including other and unknown; where the primary cancers seen at SKCCC across the 10-year period were digestive (16 555 patients), male genital (15 320 patients), and breast (14 069 patients). Regarding class of case, the highest number of patients only received treatment at SKCCC, closely followed by those who received both a diagnosis and treatment, with an increase in those who only received treatment at SKCCC between 2015 and 2019. Of all patients, 13 531 were not treated for their cancer at SKCCC, 63 302 (67.34%) had surgery, with increases in the number of patients who received radiation, chemotherapy, hormone therapy, and, especially, immunotherapy between 2015-2019. The majority of patients had private insurance, followed by Medicare and Medicaid; there were 1103 patients who had no insurance (1.17%). Finally, eTable 2 in Supplement 1 provides race and ethnicity stratified by cancer stage and eTable 3 in Supplement 1 shows race and ethnicity stratified by insurance status. Late-stage cancers increased among patients of all races and ethnicities in 2015-2019, whereas unknown stage cancers generally decreased, especially among non-Hispanic White patients. There were no striking differences in insurance type by race and ethnicity, with a slight increase in those with Medicare in 2015 to 2019.

**Table 1.** Sidney Kimmel Comprehensive Care Center Patient Characteristics Stratified by Catchment Area Between 2010 and 2019

Variable	Participants by percentage in catchment area, No. (%) (N = 94 007)			
	75% (n = 65 439)	>75%-95% (n = 17 168)	Outside 95% (n = 11 400)	Total
Cancer stage				
Early	31 796 (48.59)	8871 (51.67)	6577 (57.69)	47 244 (50.26)
Late	13 760 (21.03)	3634 (21.17)	2097 (18.39)	19 491 (20.73)
Unknown	19 883 (30.38)	4662 (27.16)	2726 (23.91)	27 271 (29.01)
Sex				
Male	32 199 (49.20)	8529 (49.68)	5281 (46.32)	46.009 (48.94)
Female	33 240 (50.80)	8639 (50.32)	6119 (53.68)	47.998 (51.06)
Age, y				
<22	748 (1.14)	217 (1.26)	86 (0.75)	1051 (1.12)
22-45	5026 (7.68)	1440 (8.39)	744 (6.53)	7210 (7.67)
46-65	20 811 (31.80)	5964 (34.74)	3420 (30.00)	30 195 (32.12)
66-75	18 676 (28.54)	5287 (30.80)	4062 (35.63)	28 025 (29.81)
>75	20 178 (30.83)	4260 (24.81)	3088 (27.09)	27 526 (29.28)
Race and ethnicity				
Asian	3572 (5.46)	369 (2.15)	268 (2.35)	4209 (4.48)
Hispanic	1991 (3.04)	237 (1.38)	180 (1.58)	2408 (2.56)
Native American	79 (0.12)	27 (0.16)	13 (0.11)	119 (0.13)
Non-Hispanic Black	14 098 (21.54)	1261 (7.35)	645 (5.66)	16 004 (17.02)
Non-Hispanic White	43 876 (67.05)	15 042 (87.62)	10 134 (88.89)	69 052 (73.45)
Other^a^	1189 (1.82)	167 (0.97)	121 (1.06)	1477 (1.57)
Unknown	634 (0.97)	65 (0.38)	39 (0.34)	738 (0.79)
Class of case				
Diagnosis and treatment	28 463 (38.05)	4783 (24.41)	2392 (18.32)	35 638 (33.17)
Diagnosis only	4295 (5.74)	530 (2.70)	343 (2.63)	5168 (4.81)
Treatment only	5430 (7.26)	1781 (9.09)	1346 (10.31)	8557 (7.96)
Nonanalytical	27 161 (36.31)	10 074 (51.41)	7319 (56.06)	44 554 (41.47)
No treatment	9446 (12.63)	2429 (12.39)	1656 (12.68)	13 531 (12.59)
Insurance				
Private	35 055 (55.04)	9846 (57.66)	6496 (57.31)	51 397 (55.80)
Medicaid	1866 (2.93)	255 (1.49)	65 (0.57)	2186 (2.37)
Medicare	22 903 (35.96)	5636 (33)	3896 (34.37)	32 435 (35.22)
Tricare	1266 (1.99)	532 (3.12)	228 (2.01)	2026 (2.20)
None	865 (1.36)	138 (0.81)	100 (0.88)	1103 (1.20)
Other	60 (0.09)	25 (0.15)	11 (0.10)	96 (0.10)
Unknown	1678 (2.63)	645 (3.78)	538 (4.75)	2861 (3.11)

^a^ Other race and ethnicity includes any other race and ethnicity not otherwise specified.

We provide the results of the full multinomial logistic regression model with interaction terms in Table 2, with the temporal cross-sections (2010-2014 vs 2015-2019) as a dummy variable. For the outcome, early-stage is the reference category with results provided for unknown and late-stage cancers. Results of the separate cross-sectional models can be found in eTable 4 and eTable 5 in Supplement 1, which contain similar results as the full model. Compared with living within the 75% CA, living outside of the 95% zone was associated with lower odds of late-stage cancer (OR, 0.72; 95% CI, 0.63-0.82). Other factors associated with decreased odds of late-stage cancer included being between 22 and 45 years of age (OR, 0.69; 95% CI, 0.63-0.76), female sex (OR, 0.88; 95% CI, 0.84-0.91), no tobacco use (OR, 0.76; 95% CI, 0.71-0.80), no alcohol use (OR, 0.94; 95% CI, 0.90-0.98), having Tricare insurance (OR, 0.83; 95% CI, 0.72-0.94), and having private insurance (OR, 0.92; 95% CI, 0.88-0.97). Compared with non-Hispanic White patients, Non-Hispanic Black patients were at increased risk of late-stage cancer (OR, 1.16; 95% CI, 1.10-1.23). Compared with those with Medicare, those with Medicaid (OR, 1.65; 95% CI, 1.46-1.86) and no insurance (OR, 2.12 95% CI, 1.79-2.51) had significantly higher odds of late-stage cancer. Regarding cancer type, chronic lymphocytic leukemia or lymphoma (OR, 1.53; 95% CI, 1.39-1.68) and respiratory cancers (OR, 1.54; 95% CI, 1.45-1.64) were associated with higher odds of a late-stage diagnosis, while breast, male genital, skin, and urinary cancers were more likely to be early-stage at diagnosis. Patients with late-stage cancers were more likely to have received immunotherapy (OR for no immunotherapy, 0.70; 95% CI, 0.65-0.75) and hormone therapy (OR for no hormone therapy, 0.76; 95% CI, 0.72-0.80). Nonanalytical patients had lower odds of late-stage cancers (OR, 0.52; 95% CI, 0.47-0.58), while patients who only received treatment at SKCCC (OR, 1.13; 95% CI, 1.08-1.19) and only received a diagnosis at SKCCC (OR, 1.26; 95% CI, 1.15-1.39) had higher odds of receiving a diagnosis of late-stage cancer. Compared with those who received a diagnosis in 2010-2014, patients who received a diagnosis between 2015 and 2019 had higher odds of late-stage cancer (OR, 1.11; 95% CI, 1.07-1.16).

**Table 2.** Multinomial Logistic Regression Results (Multivariable) for Full Sidney Kimmel Comprehensive Care Center Patient Cohort

Variable	Patient cancer stage			
	Unknown stage, OR (95% CI)	*P* value	Late-stage, OR (95% CI)	*P* value
Zone, % in catchment area				
75%	1 [Reference]	NA	1 [Reference]	NA
>75%-95%	0.97 (0.89-1.06)	.50	0.79 (0.72-0.87)	<.001
>95%	1.07 (0.95-1.20)	.30	0.72 (0.63-0.82)	<.001
Age, y				
>75	1 [Reference]	NA	1 [Reference]	NA
<22	3.32 (2.72-4.05)	<.001	0.78 (0.59-1.04)	.08
22-45	0.96 (0.88-1.05)	.30	0.69 (0.63-0.76)	<.001
46-65	0.92 (0.85-0.98)	.01	0.96 (0.90-1.02)	.20
66-75	0.87 (0.81-0.92)	<.001	0.94 (0.89-0.99)	.02
Race and ethnicity				
Non-Hispanic White	1 [Reference]	NA	1 [Reference]	NA
Asian	0.82 (0.74-0.92)	<.001	0.95 (0.86-1.05)	.30
Hispanic	0.99 (0.86-1.14)	.80	0.98 (0.86-1.13)	.80
Non-Hispanic Black	1.24 (1.17-1.32)	<.001	1.16 (1.10-1.23)	<.001
Other^a^	1.28 (1.09-1.51)	.003	0.94 (0.80-1.12)	.50
Unknown	1.86 (1.39-2.51)	<.001	1.02 (0.75-1.37)	>.90
Sex				
Male	1 [Reference]	NA	1 [Reference]	NA
Female	0.94 (0.91-0.98)	.006	0.88 (0.84-0.91)	<.001
Tobacco use				
Yes	1 [Reference]	NA	1 [Reference]	NA
No	1.08 (1.00-1.17)	.04	0.76 (0.71-0.80)	<.001
Alcohol use				
Yes	1 [Reference]	NA	1 [Reference]	NA
No	1.02 (0.97-1.07)	0.40	0.94 (0.90-0.98)	0.003
Surgery				
Yes	1 [Reference]	NA	1 [Reference]	NA
No	0.84 (0.79-0.90)	<.001	1.83 (1.74-1.92)	<.001
Radiation				
Yes	1 [Reference]	NA	1 [Reference]	NA
No	1.04 (0.99-1.10)	.11	0.98 (0.93-1.02)	.30
Chemotherapy				
Yes	1 [Reference]	NA	1 [Reference]	NA
No	0.75 (0.71-0.79)	<.001	0.59 (0.56-0.62)	<.001
Hormone therapy				
Yes	1 [Reference]	NA	1 [Reference]	NA
No	1.35 (1.27-1.43)	<.001	0.76 (0.72-0.80)	<.001
Immunotherapy				
Yes	1 [Reference]	NA	1 [Reference]	NA
No	1.07 (0.97-1.17)	.20	0.70 (0.65-0.75)	<.001
No treatment				
No	1 [Reference]	NA	1 [Reference]	NA
Yes	1.75 (1.61-1.91)	<.001	1.05 (0.97-1.12)	.20
Class of case				
Diagnosis and treatment	1 [Reference]	NA	1 [Reference]	NA
Diagnosis only	1.33 (1.20-1.48)	<.001	1.26 (1.15-1.39)	<.001
Nonanalytical	0.81 (0.74-0.90)	<.001	0.52 (0.47-0.58)	<.001
Treatment only	0.77 (0.73-0.81)	<.001	1.13 (1.08-1.19)	<.001
Cancer site				
Digestive	1 [Reference]	NA	1 [Reference]	NA
Breast	0.18 (0.16-0.20)	<.001	0.16 (0.14-0.17)	<.001
Chronic lymphocytic leukemia and lymphoma	20.10 (18.4-22.0)	<.001	1.53 (1.39-1.68)	<.001
Male genital	0.20 (0.18-0.22)	<.001	0.22 (0.21-0.23)	<.001
Other	6.24 (5.86-6.64)	<.001	0.72 (0.68-0.77)	<.001
Respiratory	0.84 (0.76-0.93)	<.001	1.54 (1.45-1.64)	<.001
Skin	0.69 (0.63-0.77)	<.001	0.26 (0.24-0.29)	<.001
Urinary	0.87 (0.79-0.95)	.001	0.29 (0.27-0.32)	<.001
Insurance				
Medicare	1 [Reference]	NA	1 [Reference]	NA
Medicaid	0.98 (0.85-1.13)	.80	1.65 (1.46-1.86)	<.001
None	1.47 (1.22-1.79)	<.001	2.12 (1.79-2.51)	<.001
Other	1.06 (0.58-1.92)	.90	0.85 (0.46-1.55)	.60
Tricare	1.00 (0.87-1.15)	>.90	0.83 (0.72-0.94)	.005
Unknown	1.89 (1.67-2.14)	<.001	1.47 (1.29-1.67)	<.001
Private	0.99 (0.94-1.05)	.80	0.92 (0.88-0.97)	.002
Year of diagnosis				
2010-2014	1 [Reference]	NA	1 [Reference]	NA
2015-2019	1.14 (1.09-1.19)	<.001	1.11 (1.07-1.16)	<.001
Zone × race and ethnicity				
>75%-95% × Asian	1.04 (0.76-1.43)	.80	0.75 (0.55-1.03)	.07
>95% × Asian	1.72 (1.18-2.50)	.005	1.92 (1.36-2.73)	<.001
>75%-95% × Hispanic	1.45 (0.97-2.15)	.06	0.83 (0.55-1.28)	.40
>95% × Hispanic	1.01 (0.63-1.63)	>.90	0.96 (0.61-1.51)	.90
>75%-95% × Non-Hispanic Black	1.07 (0.89-1.29)	.50	0.97 (0.82-1.16)	.80
>95% × Non-Hispanic Black	0.95 (0.73-1.23)	.70	1.06 (0.84-1.33)	.60
>75%-95% × other^a^	1.08 (0.70-1.66)	.70	0.80 (0.50-1.29)	.40
>95% × other^a^	1.33 (0.78-2.26)	.30	1.32 (0.75-2.30)	.30
>75%-95% × unknown	0.73 (0.33-1.62)	.40	1.47 (0.65-3.32)	.40
>95% × unknown	1.70 (0.63-4.60)	.30	1.00 (0.31-3.21)	>.90
Zone × class of case				
>75%-95% × diagnosis only	0.97 (0.71-1.31)	.80	1.34 (1.04-1.74)	.025
>95% × diagnosis only	0.72 (0.50-1.05)	.09	1.50 (1.10-2.05)	.01
>75%-95% × nonanalytical	0.86 (0.72-1.03)	.11	0.78 (0.63-0.97)	.02
>95% × nonanalytical	1.00 (0.81-1.24)	>.90	0.95 (0.75-1.21)	.70
>75%-95% × treatment only	0.97 (0.87-1.09)	.60	1.44 (1.28-1.61)	<.001
>95% × treatment only	0.96 (0.83-1.11)	.60	1.18 (1.02-1.36)	.03

Abbreviation: NA, not applicable; OR, odds ratio.

^a^ Other race and ethnicity includes any other race and ethnicity not otherwise specified.

The interaction terms yielded statistically significant findings for both CA and zone by race and ethnicity and CA and zone by class of case. Asian patients residing outside the 95% zone had higher odds of late-stage cancers (OR, 1.92; 95% CI, 1.36-2.73). Patients who received only a diagnosis at SKCCC and were residing in the greater than 75% to 95% zone (OR, 1.34; 95% CI, 1.04-1.74) or outside the 95% zone (OR, 1.50; 95% CI, 1.10-2.05) had higher odds of late-stage cancers. Those who only received treatment at SKCCC and were residing in the greater than 75% to 95% zone (OR, 1.44; 95% CI, 1.28-1.61) or outside the 95% zone (OR, 1.18; 95% CI, 1.02-1.36) also had higher odds of late-stage cancers. Finally, nonanalytical cases residing in the greater than 75% to 95% zone had lower odds of late-stage cancers (OR, 0.78; 95% CI, 0.63-0.97).

## Discussion

To our knowledge, this cross-sectional study is one of the most detailed analyses of an NCI CCC across a decade of patient registry data and service to patients. We believe another major contribution of this study is the use of geospatial techniques to define and evaluate cancer center CAs in a way that is easily reproducible for other facilities evaluating patient utilization and outcomes to improve research programs and mitigate late and unknown cancer staging, thereby improving survival, especially among those most vulnerable. We encourage others to update their CAs over time to better capture the changing patient dynamics, such as utilization, cancer screening, and treatment needs. Notably, we did not find evidence of geographic disparities in late-stage cancers, in general, for patients living in the greater than 75% to 95% zone and outside the 95% zone, except for Asian patients, those who only received treatment at SKCCC, and those who were only diagnosed at SKCCC. We also found evidence that late-stage cancers were more prevalent among the 2015 to 2019 cohort, while newer and modern treatments including hormone and especially immunotherapy also increased in this time frame.

A major additional finding was that having no insurance, unknown insurance, or Medicaid was associated with higher odds of receiving a diagnosis of late-stage cancer. While Medicaid covers several screening and prevention services, many individuals will not secure coverage until they face a cancer diagnosis due to the subsequent medical bills. State Medicaid programs have contractual entanglements; therefore, Medicaid programs and the NCI cancer centers should work together to improve access and utilization of cancer risk reduction, screening, and early detection services. This finding also underscores the challenges that low-income and socially disadvantaged individuals face despite increasing access to health insurance through Medicaid expansion^26^ and highlights the need to better understand social determinants of health (SDoH), delays in cancer screening, lifestyle and behavior factors (eg, substance use), environmental exposures, and injustices.^27^

The second major finding was that non-Hispanic Black patients were at an increased risk of receiving a diagnosis of late-stage cancers, regardless of proximity to SKCCC. This finding aligns with other studies^28,29,30^ that found lower odds of survival for non-Hispanic Black patients. Studies^31,32,33^ have also shown that non-Hispanic Black patients may also experience lower rates of screening and longer follow-up times, leading to higher rates of late-stage cancers at diagnosis. Furthermore, many non-Hispanic Black patients in our study resided near SKCCC; therefore, accessibility is more complex than distance-to-care or screening facilities. This finding further supports better capture of the SDoH to reduce racial and ethnic health disparities.

The third and most striking finding was that geographic disparities in late-stage cancers for patients who received only treatment or only received a diagnosis at SKCCC were observed in the outer zones or outside the 95% zone altogether. The substantial distances traveled may be a factor for seeking partial services (ie, only diagnosis or only treatment). The expert services sought may have been specific to SKCCC, given the distances involved. This finding suggests that many SKCCC patients will share their cancer care (ie, receive care at more than 1 cancer center or facility). Those who only received a diagnosis at SKCCC may have been seen for a second opinion, moved before treatment, or visited a CCC for trust in diagnostic procedures. Those who only received treatment at SKCCC may have been coming for specialized treatment (eg, immunotherapy), clinical trials, moved after diagnosis, or sought care for certain cancer types (eg, blood, prostate, ovarian, and lung). Therefore, screening and treatment options should be improved throughout the US, regardless of CCC attendance, and we recommend both diagnosis and treatment should both occur at a CCC, if possible. The other geographic disparity was among Asian patients outside of the 95% zone. This finding corroborates other findings^34,35,36^ that suggest that this subpopulation may have a generally higher socioeconomic status, especially those that are US-born. However, despite being the highest-earning socioeconomic group in the US, the income-gap among Asians has grown multifold in recent decades.^37,38^ Therefore, SDoH is again an important factor when studying cancer outcomes, irrespective of geography and race or ethnicity.

We believe our approach can help with the following. First, this approach can help identify individuals and areas that experience a high degree of care-sharing. Our findings showed that those who only received treatment or those who only received a diagnosis at our CCC outside of the main CA had higher odds of being diagnosed with late-stage cancer. This finding suggests that patients may travel farther distances to seek higher quality diagnostic resources or for specialized treatment for late-stage cancer. Because patients that received both diagnosis and treatment at SKCCC had lower odds of a late-stage diagnosis, we believe this is an opportunity for all CCCs to collaborate on optimizing care-sharing models to improve screening and treatment outcomes (because 1 CCC may not have the resources or specialties to both diagnose and treat a particular cancer). We do not know where our patients received treatment or a diagnosis before or after utilizing our CCC. We envision that each CCC could identify their closest 75% of patients (using our spatial approach here) and then identify which facility their patients received a cancer diagnosis or where they went to get treated. Second, at the patient level, our approach could be used as a national-level dashboard or tool that could show the CA of each CCC, overlap of multiple CCCs, what cancers each CCC treats, and which CCC is worth traveling to for resources that maximize their outcomes and survival. Areas with a high-proportion of care-sharing could be targeted to determine if and why late-stage diagnoses are more common (eg, proximity to a CCC, various health disparities, SDoH, poor access to primary care, and delays in screening).

### Limitations

Despite the strengths of our research, we acknowledge several limitations. First, we did not consider travel time when computing the CAs of closest patients. Future research can consider travel time, which may also differ by modes of transportation. However, these data were not available with the cohort in this study. Future studies can also request finer-level data to highlight potential within-ZCTA variation, such as neighborhood-level characteristics. Next, we did not examine the association of late-stage cancer with subsequent death and overall survival. Of note, the registry data did not indicate if a patient’s death was related to cancer or another cause. Third, we did not adjust for comorbidities, which was beyond the scope of this research. Fourth, we did not explicitly account for the differences in Medicaid expansion; for example, Virginia expanded Medicaid in 2019 and Pennsylvania expanded in 2015. Fifth, we did not have residential histories of the patients which would better capture exposures of patients before receiving a diagnosis at the ZCTA listed in the current SKCCC registry. Sixth, this population-based study did not capture the nuances of cancer diagnoses and care, such as health seeking behaviors, perspectives, knowledge, and other barriers which can be collected in a mixed-methods approach. Seventh, our cohort of patients was studied before the COVID-19 pandemic, which may have exacerbated cancer screening, care, and outcomes for certain populations.^39,40,41^

## Conclusions

In this cross-sectional study of patient utilization data for a CCC across a decade, we found that patients residing outside the main CA who received only treatment or only a diagnosis at SKCCC had higher odds of a late-stage cancer diagnosis. Racial disparities in staging were evident for non-Hispanic Black patients, regardless of geographic proximity. Those with Medicaid or no or unknown insurance had significantly higher odds of late-stage cancers, regardless of race and ethnicity or distance from SKCCC. These findings indicate that cancer outcomes have not improved for disadvantaged populations utilizing SKCCC for cancer care in our 10-year study period. CCCs should improve surveillance of SDoH to better capture nuances in disparities in cancer stage for their patients and update their CAs over time to account for varying patient utilization and cancer surveillance and outcomes. Finally, NCI designation of a CCC only reflects successful receipt of a grant that supports research infrastructure; centers should more actively consider their service areas in terms of health care needs, and geospatial analyses could facilitate the prioritization of improved services.

## Notes

Supplement 1. **eTable 1.** Full Descriptive Statistics of SKCCC Cancer Patients Stratified by Zone (75% CA->95%) and Year 2010-2014 & 2015-2019 **eTable 2**. Descriptive Statistics of Cancer Staging by Race/Ethnicity for SKCCC Patients (2010-2014 & 2015-2019) **eTable 3.** Descriptive Statistics of Insurance Status by Race/Ethnicity for SKCCC Patients (2010-2014 & 2015-2019) **eTable 4.** Multinomial LR Results (Multivariable) for 2010-2014 SKCCC Patient Cohort **eTable 5.** Multinomial LR Results for 2015-2019 SKCCC Patient Cohort

Supplement 2.
Data Sharing Statement
