# Supplementary material for: Disparities in Cancer Stage Outcomes by Catchment Areas for a Comprehensive Cancer Center
Source: JAMA Netw Open. 2024 May 2;7(5):e249474. doi: 10.1001/jamanetworkopen.2024.9474 (PMC11066700; doi:10.1001/jamanetworkopen.2024.9474)
Supplement: Supplement 1. — eTable 1. Full Descriptive Statistics of SKCCC Cancer Patients Stratified by Zone (75% CA->95%) and Year 2010-2014 & 2015-2019 eTable 2. Descriptive Statistics of Cancer Staging by Race/Ethnicity for SKCCC Patients (2010-2014 & 2015-2019) eTable 3. Descriptive Statistics of Insurance Status by Race/Ethnicity for SKCCC Patients (2010-2014 & 2015-2019) eTable 4. Multinomial LR Results (Multivariable) for 2010-2014 SKCCC Patient Cohort eTable 5. Multinomial LR Results for 2015-2019 SKCCC Patient Cohort [file jamanetwopen-e249474-s001.pdf]

Supplemental Online Content

Desjardins MR, Kanarek NF, Nelson WG, Bachman J, Curriero FC. Disparities in cancer stage outcome by catchment areas for a comprehensive cancer center. *JAMA Netw Open*. 2024;7(5):e249474. doi:10.1001/jamanetworkopen.2024.9474

eTable 1. Full Descriptive Statistics of SKCCC Cancer Patients Stratified bv Zone (75% CA->95%) and Year 2010-2014 & 2015-2019

eTable 2. Descriptive Statistics of Cancer Staging by Race/Ethnicity for SKCCC Patients (2010-2014 & 2015-2019)

eTable 3. Descriptive Statistics of Insurance Status by Race/Ethnicity for SKCCC Patients (2010-2014 & 2015-2019)

eTable 4. Multinomial LR Results (Multivariate) for 2010-2014 SKCCC Patient Cohort

eTable 5. Multinomial LR Results for 2015-2019 SKCCC Patient Cohort

This supplemental material has been provided by the authors to give readers additional information about their work.

© 2024 Desjardins MR et al. *JAMA Network Open.*

eTable 1. Full Descriptive Statistics of SKCCC Cancer Patients Stratified bv Zone (75% CA->95%) and Year 2010-2014 & 2015-2019

Variable (Years)	75% CA	>75%-80% Zone	85% Zone	90% Zone	95% Zone	>95% Zone	Total
**Cancer Stage**	No. (%)	No. (%)	No. (%)	No. (%)	No. (%)	No. (%)	No. (%)
Early (10-14)	14680 (46.87)	1051 (49.83)	1083 (49.63)	1274 (52.19)	1149 (51.76)	3904 (58.04)	23141 (49.23)
Late (10-14)	5742 (18.33)	373 (17.69)	414 (18.97)	468 (19.17)	455 (20.5)	1071 (15.92)	8523 (18.13)
Unknown (10-14)	10902 (34.8)	685 (32.48)	685 (31.39)	699 (28.64)	616 (27.75)	1751 (26.03)	15338 (32.63)
Early (15-19)	17116 (50.17)	1106 (52.19)	1009 (50.5)	1085 (55.08)	1114 (52.35)	2673 (57.19)	24103 (51.28)
Late (15-19)	8018 (23.5)	455 (21.47)	489 (24.47)	443 (22.49)	537 (25.23)	1026 (21.95)	10968 (23.33)
Unknown (15-19)	8981 (26.33)	558 (26.33)	500 (25.03)	442 (22.44)	477 (22.42)	975 (20.86)	11933 (25.39)
**Sex**	No. (%)	No. (%)	No. (%)	No. (%)	No. (%)	No. (%)	No. (%)
Male (10-14)	16324 (52.11)	991 (46.97)	986 (45.19)	1030 (42.2)	858 (38.65)	2336 (34.73)	22525 (47.92)
Female (10-14)	15000 (47.89)	1119 (53.03)	1196 (54.81)	1411 (57.8)	1362 (61.35)	4390 (65.27)	24478 (52.08)
Male (15-19)	15875 (46.53)	1153 (54.41)	1102 (55.16)	1122 (56.95)	1287 (60.48)	2945 (63.01)	23484 (49.96)
Female (15-19)	18240 (53.47)	966 (45.59)	896 (44.84)	848 (43.05)	841 (39.52)	1729 (36.99)	23520 (50.04)
**Age**	No. (%)	No. (%)	No. (%)	No. (%)	No. (%)	No. (%)	No. (%)
<22 (10-14)	271 (0.87)	25 (1.2)	36 (1.66)	30 (1.24)	14 (0.64)	50 (0.75)	426 (0.91)
22-45 (10-14)	1912 (6.16)	202 (9.67)	171 (7.89)	172 (7.1)	122 (5.54)	399 (5.95)	2978 (6.39)
46-65 (10-14)	8751 (28.18)	742 (35.54)	679 (31.35)	746 (30.79)	621 (28.19)	1825 (27.23)	13364 (28.66)
66-75 (10-14)	8899 (28.66)	587 (28.11)	643 (29.69)	759 (31.32)	737 (33.45)	2329 (34.76)	13954 (29.92)
>75 (10-14)	11216 (36.12)	532 (25.48)	637 (29.41)	716 (29.55)	709 (32.18)	2098 (31.31)	15908 (34.12)
<22 (15-19)	477 (1.39)	36 (1.68)	28 (1.39)	16 (0.8)	32 (1.49)	36 (0.77)	625 (1.32)
22-45 (15-19)	3114 (9.05)	228 (10.65)	203 (10.08)	185 (9.31)	157 (7.32)	345 (7.34)	4232 (8.93)
46-65 (15-19)	12060 (35.07)	879 (41.06)	799 (39.67)	761 (38.28)	737 (34.36)	1595 (33.94)	16831 (35.53)

© 2024 Desjardins MR et al. *JAMA Network Open.*

66-75 (15-19)	9777 (28.43)	579 (27.04)	611 (30.34)	625 (31.44)	746 (34.78)	1733 (36.88)	14071 (29.7)
>75 (15-19)	8962 (26.06)	419 (19.57)	373 (18.52)	401 (20.17)	473 (22.05)	990 (21.07)	11618 (24.52)
**Race**	No. (%)	No. (%)	No. (%)	No. (%)	No. (%)	No. (%)	No. (%)
Non-Hisp White (10-14)	21521 (68.7)	1711 (81.09)	1979 (90.7)	2203 (90.25)	1991 (89.68)	6018 (89.47)	35423 (75.36)
Non-Hisp Black (10-14)	6605 (21.09)	216 (10.24)	132 (6.05)	160 (6.55)	138 (6.22)	380 (5.65)	7631 (16.24)
Native American (10-14)	35 (0.11)	3 (0.14)	1 (0.05)	2 (0.08)	2 (0.09)	7 (0.1)	50 (0.11)
Asian (10-14)	1455 (4.65)	85 (4.03)	23 (1.05)	32 (1.31)	33 (1.49)	151 (2.25)	1779 (3.78)
Other (10-14)	562 (1.79)	31 (1.47)	19 (0.87)	14 (0.57)	25 (1.13)	64 (0.95)	715 (1.52)
Unknown (10-14)	320 (1.02)	10 (0.47)	5 (0.23)	6 (0.25)	11 (0.5)	18 (0.27)	370 (0.79)
Hispanic (10-14)	826 (2.64)	54 (2.56)	23 (1.05)	24 (0.98)	20 (0.9)	88 (1.31)	1035 (2.2)
Non-Hisp White (15-19)	22355 (65.53)	1749 (82.54)	1823 (91.24)	1732 (87.92)	1854 (87.12)	4116 (88.06)	33629 (71.54)
Non-Hisp Black (15-19)	7493 (21.96)	190 (8.97)	92 (4.6)	163 (8.27)	170 (7.99)	265 (5.67)	8373 (17.81)
Native American (15-19)	44 (0.13)	4 (0.19)	6 (0.3)	4 (0.2)	5 (0.23)	6 (0.13)	69 (0.15)
Asian (15-19)	2117 (6.21)	97 (4.58)	24 (1.2)	35 (1.78)	40 (1.88)	117 (2.5)	2430 (5.17)
Other (15-19)	627 (1.84)	33 (1.56)	16 (0.8)	10 (0.51)	19 (0.89)	57 (1.22)	762 (1.62)
Unknown (15-19)	314 (0.92)	8 (0.38)	10 (0.5)	6 (0.3)	9 (0.42)	21 (0.45)	368 (0.78)
Hispanic (15-19)	1165 (3.41)	38 (1.79)	27 (1.35)	20 (1.02)	31 (1.46)	92 (1.97)	1373 (2.92)
**Class of Case**	No. (%)	No. (%)	No. (%)	No. (%)	No. (%)	No. (%)	No. (%)
Dx & Treated (10-14)	15115 (42.51)	707 (29.89)	760 (30.56)	687 (24.85)	622 (24.76)	1504 (19.75)	19395 (36.39)
Dx Only (10-14)	1959 (5.51)	71 (3)	59 (2.37)	64 (2.31)	62 (2.47)	206 (2.71)	2421 (4.54)
Non-Analytical (10-14)	2268 (6.38)	215 (9.09)	231 (9.29)	268 (9.69)	271 (10.79)	839 (11.02)	4092 (7.68)
Treat Only (10-14)	11982 (33.7)	1117 (47.23)	1132 (45.52)	1422 (51.43)	1265 (50.36)	4177 (54.85)	21095 (39.58)
No Treatment (10-14)	4232 (11.9)	255 (10.78)	305 (12.26)	324 (11.72)	292 (11.62)	889 (11.67)	6297 (11.81)
Dx & Treated (15-19)	13348 (34.02)	603 (24.81)	507 (22.01)	431 (19.04)	466 (18.87)	888 (16.32)	16243 (30)
Dx Only (15-19)	2336 (5.95)	63 (2.59)	67 (2.91)	58 (2.56)	86 (3.48)	137 (2.52)	2747 (5.07)
Non-Analytical (15-19)	3162 (8.06)	200 (8.23)	169 (7.34)	213 (9.41)	214 (8.66)	507 (9.32)	4465 (8.25)
Treat Only (15-19)	15179 (38.68)	1253 (51.56)	1255 (54.47)	1268 (56.01)	1362 (55.14)	3142 (57.75)	23459 (43.32)

© 2024 Desjardins MR et al. *JAMA Network Open.*

No Treatment (15-19)	5214 (13.29)	311 (12.8)	306 (13.28)	294 (12.99)	342 (13.85)	767 (14.1)	7234 (13.36)
**Surgery**	No. (%)	No. (%)	No. (%)	No. (%)	No. (%)	No. (%)	No. (%)
2010-2014	20643 (65.51)	1460 (4.63)	1521 (4.83)	1711 (5.43)	1545 (4.9)	4629 (14.69)	31509 (100)
2015-2019	22826 (71.8)	1497 (4.71)	1379 (4.34)	1393 (4.38)	1436 (4.52)	3262 (10.26)	31793 (100)
**Radiation**	No. (%)	No. (%)	No. (%)	No. (%)	No. (%)	No. (%)	No. (%)
2010-2014	7492 (68.71)	505 (4.63)	517 (4.74)	528 (4.84)	482 (4.42)	1380 (12.66)	10904 (47.5)
2015-2019	9250 (76.76)	447 (3.71)	447 (3.71)	448 (3.72)	509 (4.22)	949 (7.88)	12050 (52.5)
**Chemo**	No. (%)	No. (%)	No. (%)	No. (%)	No. (%)	No. (%)	No. (%)
2010-2014	9813 (71.12)	601 (4.36)	604 (4.38)	653 (4.73)	577 (4.18)	1549 (11.23)	13797 (47.58)
2015-2019	11196 (73.66)	673 (4.43)	641 (4.22)	605 (3.98)	693 (4.56)	1392 (9.16)	15200 (52.42)
**Immunotherapy**	No. (%)	No. (%)	No. (%)	No. (%)	No. (%)	No. (%)	No. (%)
2010-2014	1040 (67.18)	59 (3.81)	76 (4.91)	81 (5.23)	75 (4.84)	217 (14.02)	1548 (26.38)
2015-2019	3578 (82.8)	143 (3.31)	133 (3.08)	101 (2.34)	144 (3.33)	222 (5.14)	4321 (73.62)
**Hormone**	No. (%)	No. (%)	No. (%)	No. (%)	No. (%)	No. (%)	No. (%)
2010-2014	4348 (66.11)	363 (5.52)	355 (5.4)	373 (5.67)	316 (4.8)	822 (12.5)	6577 (45.39)
2015-2019	5572 (70.41)	354 (4.47)	369 (4.66)	348 (4.4)	388 (4.9)	883 (11.16)	7914 (54.61)
**Cancer Site**	No. (%)	No. (%)	No. (%)	No. (%)	No. (%)	No. (%)	No. (%)
Bones & Joints (10-14)	128 (0.41)	19 (0.91)	8 (0.37)	21 (0.87)	14 (0.64)	51 (0.76)	241 (0.52)
Brain & ONS (10-14)	1847 (5.95)	159 (7.61)	186 (8.59)	191 (7.88)	183 (8.31)	644 (9.61)	3210 (6.88)
Breast (10-14)	5498 (17.71)	218 (10.44)	190 (8.77)	210 (8.67)	161 (7.31)	388 (5.79)	6665 (14.29)
Digestive (10-14)	5057 (16.29)	276 (13.22)	356 (16.44)	401 (16.55)	455 (20.65)	1615 (24.1)	8160 (17.5)
Endocrine (10-14)	1859 (5.99)	254 (12.16)	217 (10.02)	155 (6.4)	123 (5.58)	217 (3.24)	2825 (6.06)
Eye & Orb (10-14)	144 (0.46)	24 (1.15)	15 (0.69)	21 (0.87)	11 (0.5)	29 (0.43)	244 (0.52)
Female Repro (10-14)	1465 (4.72)	62 (2.97)	58 (2.68)	67 (2.77)	53 (2.41)	107 (1.6)	1812 (3.89)
Heart & Soft Tiss (10-14)	273 (0.88)	32 (1.53)	34 (1.57)	33 (1.36)	37 (1.68)	53 (0.79)	462 (0.99)
Leukemia (10-14)	1507 (4.85)	129 (6.18)	155 (7.16)	156 (6.44)	114 (5.17)	234 (3.49)	2295 (4.92)
Lymphoma (10-14)	927 (2.99)	66 (3.16)	61 (2.82)	57 (2.35)	51 (2.32)	86 (1.28)	1248 (2.68)
Male Genital (10-14)	4322 (13.92)	328 (15.71)	361 (16.67)	455 (18.78)	482 (21.88)	2132 (31.82)	8080 (17.33)

© 2024 Desjardins MR et al. *JAMA Network Open.*

Oral & Phar (10-14)	713 (2.3)	79 (3.78)	67 (3.09)	93 (3.84)	104 (4.72)	161 (2.4)	1217 (2.61)
Other (10-14)	25 (0.08)	1 (0.05)	2 (0.09)	0 (0)	2 (0.09)	3 (0.04)	33 (0.07)
Respiratory (10-14)	3031 (9.76)	138 (6.61)	149 (6.88)	191 (7.88)	157 (7.13)	337 (5.03)	4003 (8.58)
Skin (10-14)	1748 (5.63)	134 (6.42)	121 (5.59)	135 (5.57)	86 (3.9)	164 (2.45)	2388 (5.12)
Unknown (10-14)	321 (1.03)	11 (0.53)	13 (0.6)	11 (0.45)	13 (0.59)	31 (0.46)	400 (0.86)
Urinary (10-14)	2184 (7.03)	158 (7.57)	173 (7.99)	226 (9.33)	157 (7.13)	449 (6.7)	3347 (7.18)
Bones & Joints (15-19)	176 (0.51)	12 (0.56)	14 (0.7)	11 (0.55)	14 (0.65)	44 (0.94)	271 (0.57)
Brain & ONS (15-19)	2046 (5.95)	154 (7.19)	152 (7.55)	152 (7.65)	153 (7.13)	425 (9.04)	3082 (6.51)
Breast (15-19)	6458 (18.78)	181 (8.45)	173 (8.59)	167 (8.4)	136 (6.34)	289 (6.15)	7404 (15.63)
Digestive (15-19)	5451 (15.85)	381 (17.8)	385 (19.12)	378 (19.01)	483 (22.52)	1317 (28.03)	8395 (17.72)
Endocrine (15-19)	1755 (5.1)	187 (8.73)	154 (7.65)	114 (5.73)	95 (4.43)	162 (3.45)	2467 (5.21)
Eye & Orb (15-19)	164 (0.48)	25 (1.17)	17 (0.84)	11 (0.55)	17 (0.79)	15 (0.32)	249 (0.53)
Female Repro (15-19)	1714 (4.98)	87 (4.06)	59 (2.93)	62 (3.12)	66 (3.08)	115 (2.45)	2103 (4.44)
Heart & Soft Tiss (15-19)	420 (1.22)	31 (1.45)	36 (1.79)	31 (1.56)	42 (1.96)	40 (0.85)	600 (1.27)
Leukemia (15-19)	1792 (5.21)	147 (6.87)	126 (6.26)	108 (5.43)	105 (4.9)	145 (3.09)	2423 (5.11)
Lymphoma (15-19)	935 (2.72)	55 (2.57)	61 (3.03)	44 (2.21)	42 (1.96)	47 (1)	1184 (2.5)
Male Genital (15-19)	4379 (12.73)	335 (15.65)	322 (15.99)	399 (20.07)	431 (20.09)	1374 (29.24)	7240 (15.28)
Oral & Phar (15-19)	904 (2.63)	73 (3.41)	86 (4.27)	86 (4.33)	83 (3.87)	106 (2.26)	1338 (2.82)
Other (15-19)	17 (0.05)	2 (0.09)	2 (0.1)	1 (0.05)	0 (0)	4 (0.09)	26 (0.05)
Respiratory (15-19)	3493 (10.16)	169 (7.89)	151 (7.5)	137 (6.89)	174 (8.11)	197 (4.19)	4321 (9.12)
Skin (15-19)	1886 (5.48)	125 (5.84)	107 (5.31)	102 (5.13)	75 (3.5)	93 (1.98)	2388 (5.04)
Unknown (15-19)	320 (0.93)	7 (0.33)	11 (0.55)	0 (0)	10 (0.47)	18 (0.38)	366 (0.77)
Urinary (15-19)	2480 (7.21)	170 (7.94)	158 (7.85)	185 (9.31)	219 (10.21)	308 (6.55)	3520 (7.43)
**Insurance**	No. (%)	No. (%)	No. (%)	No. (%)	No. (%)	No. (%)	No. (%)
Medicaid (10-14)	968 (3.12)	43 (2.06)	35 (1.62)	19 (0.78)	32 (1.45)	36 (0.54)	1133 (2.43)
Medicare (10-14)	10175 (32.83)	517 (24.77)	619 (28.59)	721 (29.76)	747 (33.91)	2090 (31.2)	14869 (31.87)
None (10-14)	563 (1.82)	19 (0.91)	28 (1.29)	21 (0.87)	28 (1.27)	79 (1.18)	738 (1.58)
Other (10-14)	39 (0.13)	3 (0.14)	5 (0.23)	3 (0.12)	2 (0.09)	5 (0.07)	57 (0.12)
Tricare (10-14)	515 (1.66)	88 (4.22)	63 (2.91)	78 (3.22)	33 (1.5)	121 (1.81)	989 (2.12)

© 2024 Desjardins MR et al. *JAMA Network Open.*

Unknown (10-14)	1525 (4.92)	129 (6.18)	142 (6.56)	186 (7.68)	158 (7.17)	516 (7.7)	2656 (5.69)
Private (10-14)	17208 (55.52)	1288 (61.72)	1273 (58.8)	1395 (57.57)	1203 (54.61)	3852 (57.5)	26219 (56.19)
Medicaid (15-19)	898 (2.75)	25 (1.18)	41 (2.05)	21 (1.07)	39 (1.84)	29 (0.63)	1053 (2.31)
Medicare (15-19)	12728 (38.92)	663 (31.38)	720 (35.98)	723 (36.78)	926 (43.7)	1806 (38.96)	17565 (38.58)
None (15-19)	302 (0.92)	6 (0.28)	11 (0.55)	12 (0.61)	13 (0.61)	21 (0.45)	365 (0.8)
Other (15-19)	21 (0.06)	8 (0.38)	2 (0.1)	1 (0.05)	1 (0.05)	6 (0.13)	39 (0.09)
Tricare (15-19)	751 (2.3)	113 (5.35)	64 (3.2)	61 (3.1)	32 (1.51)	107 (2.31)	1128 (2.48)
Unknown (15-19)	153 (0.47)	8 (0.38)	11 (0.55)	6 (0.31)	5 (0.24)	22 (0.47)	205 (0.45)
Private (15-19)	17847 (54.58)	1290 (61.05)	1152 (57.57)	1142 (58.09)	1103 (52.05)	2644 (57.04)	25178 (55.3)

eTable 2. Descriptive Statistics of Cancer Staging by Race/Ethnicity for SKCCC Patients (2010-2014 & 2015-2019)

	**Early Stage**	**Late-stage**	**Unknown Stage**	**Total**
**Race/Ethnicity**	No. (%)	No. (%)	No. (%)	No. (%)
Non-Hisp White (10-14)	18067 (51.0)	6299 (17.78)	11057 (31.21)	35423 (75.36)
Non-Hisp Black (10-14)	3429 (44.94)	1576 (20.65)	2626 (34.41)	7631 (16.24)
Native American (10-14)	27 (54.0)	7 (14.0)	16 (32.0)	50 (0.11)
Asian (10-14)	776 (43.62)	341 (19.17)	662 (37.21)	1779 (3.78)
Other (10-14)	343 (47.97)	111 (15.52)	261 (36.5)	715 (1.52)
Unknown (10-14)	108 (29.19)	36 (9.73)	226 (61.08)	370 (0.79)
Hispanic (10-14)	392 (37.87)	153 (14.78)	490 (47.34)	1035 (2.2)
Non-Hisp White (15-19)	17700 (52.63)	7702 (22.9)	8227 (24.46)	33629 (71.52)
Non-Hisp Black (15-19)	4014 (47.94)	2187 (26.12)	2172 (25.94)	8373 (17.81)
Native American (15-19)	28 (40.58)	16 (23.19)	25 (36.23)	69 (0.15)
Asian (15-19)	1221 (50.25)	548 (22.55)	661 (27.2)	2430 (5.17)
Other (15-19)	363 (47.64)	159 (20.87)	240 (31.5)	762 (1.62)
Unknown (15-19)	226 (42.4)	138 (25.89)	169 (31.71)	533 (1.13)
Hispanic (15-19)	490 (40.03)	295 (24.1)	439 (35.87)	1224 (2.6)

© 2024 Desjardins MR et al. *JAMA Network Open.*

eTable 3. Descriptive Statistics of Insurance Status by Race/Ethnicity for SKCCC Patients (2010-2014 & 2015-2019)

**Insurance Type**	**Medicaid**	**Medicare**	**None**	**Other**
**Race/Ethnicity**	No. (%)	No. (%)	No. (%)	No. (%)
Non-Hisp White (10-14)	446 (39.36)	11740 (78.96)	353 (47.83)	43 (75.44)
Non-Hisp Black (10-14)	535 (47.22)	2239 (15.06)	214 (29)	7 (12.28)
Native American (10-14)	0 (0.0)	12 (0.08)	0 (0.0)	0 (0.0)
Asian (10-14)	68 (6.0)	405 (2.72)	50 (6.78)	4 (7.02)
Other (10-14)	17 (1.5)	149 (1.0)	25 (3.39)	0 (0.0)
Unknown (10-14)	7 (0.62)	105 (0.71)	7 (0.95)	0 (0.0)
Hispanic (10-14)	60 (5.3)	219 (1.47)	89 (12.06)	3 (5.26)
Non-Hisp White (15-19)	410 (39.31)	13228 (75.31)	137 (37.53)	30 (76.92)
Non-Hisp Black (15-19)	443 (42.47)	2965 (16.88)	95 (26.03)	6 (15.38)
Native American (15-19)	0 (0.0)	27 (0.15)	0 (0)	0 (0.0)
Asian (15-19)	58 (5.56)	691 (3.93)	37 (10.14)	1 (2.56)
Other (15-19)	36 (3.45)	220 (1.25)	14 (3.84)	0 (0.0)
Unknown (15-19)	10 (0.96)	97 (0.55)	11 (3.01)	0 (0.0)
Hispanic (15-19)	86 (8.25)	337 (1.92)	71 (19.45)	2 (5.13)
**Insurance Type**	**Tricare**	**Unknown**	**Private**	**Total**
**Race/Ethnicity**	No. (%)	No. (%)	No. (%)	No. (%)
Non-Hisp White (10-14)	683 (76.06)	2054 (77.33)	19785 (75.46)	35104 (75.38)
Non-Hisp Black (10-14)	158 (17.59)	366 (13.78)	4024 (15.35)	7543 (16.2)
Native American (10-14)	2 (0.22)	3 (0.11)	33 (0.13)	50 (0.11)
Asian (10-14)	20 (2.23)	102 (3.84)	1118 (4.26)	1767 (3.79)
Other (10-14)	11 (1.22)	62 (2.33)	447 (1.7)	711 (1.53)
Unknown (10-14)	6 (0.67)	17 (0.64)	226 (0.86)	368 (0.79)
Hispanic (10-14)	18 (2.0)	52 (1.96)	586 (2.24)	1027 (2.21)
Non-Hisp White (15-19)	813 (72.07)	133 (64.88)	17887 (71.01)	32638 (71.68)
Non-Hisp Black (15-19)	224 (19.86)	40 (19.51)	4427 (17.58)	8200 (18.01)
Native American (15-19)	3 (0.27)	1 (0.49)	33 (0.13)	64 (0.14)
Asian (15-19)	46 (4.08)	16 (7.8)	1429 (5.67)	2278 (5.0)
Other (15-19)	14 (1.24)	4 (1.95)	434 (1.72)	722 (1.59)

© 2024 Desjardins MR et al. *JAMA Network Open.*

Unknown (15-19)	5 (0.44)	3 (1.46)	191 (0.76)	317 (0.7)
Hispanic (15-19)	23 (2.04)	8 (3.9)	787 (3.12)	1314 (2.89)

© 2024 Desjardins MR et al. *JAMA Network Open.*

eTable 4. Multinomial LR Results (Multivariate) for 2010-2014 SKCCC Patient Cohort

**Stage at Diagnosis**	**Unknown**		**Late**	
**Variable**	**OR[1] (95% CI)**	**p-value**	**OR[1] (95% CI)**	**p-value**
**Zone**				
75% CA	—		—	
>75%-95%	0.76 (0.68-0.84)	<0.001	0.82 (0.72-0.93)	0.002
>95%	0.85 (0.75-0.98)	0.02	0.69 (0.59-0.82)	<0.001
**Age**				
>75	—		—	
<22	2.89 (2.12-3.96)	<0.001	1.02 (0.65-1.60)	>0.90
22-45	0.86 (0.77-0.96)	0.008	0.62 (0.53-0.72)	<0.001
46-65	0.9 (0.83-0.97)	0.007	0.9 (0.82-0.98)	0.02
66-75	0.87 (0.81-0.93)	<0.001	0.94 (0.86-1.02)	0.12
**Race/Ethnicity**				
Non-Hisp White	—		—	
Asian	1.04 (0.91-1.19)	0.60	0.97 (0.83-1.14)	0.70
Hispanic	1.79 (1.50-2.12)	<0.001	1.06 (0.84-1.34)	0.60
Non-Hisp Black	1.14 (1.06-1.22)	<0.001	1.25 (1.16-1.36)	<0.001
Other	0.85 (0.69-1.04)	0.11	0.87 (0.68-1.13)	0.30
Unknown	4.9 (3.73-6.42)	<0.001	1.27 (0.81-1.99)	0.30
**Female Sex**	1.32 (1.24-1.39)	<0.001	1.3 (1.22-1.38)	<0.001
**Tobacco Use**				
Yes	—		—	
No	1.35 (1.23-1.48)	<0.001	0.71 (0.65-0.77)	<0.001
**Alcohol Use**				
Yes	—		—	
No	1.49 (1.41-1.58)	<0.001	1.01 (0.94-1.07)	0.80
**Surgery**				
Yes	—		—	
No	1.21 (1.13-1.29)	<0.001	1.17 (1.08-1.27)	<0.001
**Radiation**				
Yes	—		—	
No	1 (0.95-1.06)	>0.90	1.15 (1.07-1.23)	<0.001

© 2024 Desjardins MR et al. *JAMA Network Open.*

**Chemo**				
Yes	—		—	
No	0.97 (0.91-1.03)	0.30	1.09 (1.02-1.17)	0.01
**Hormone Therapy**				
Yes	—		—	
No	1.23 (1.15-1.32)	<0.001	0.99 (0.90-1.08)	0.80
**Immunotherapy**				
Yes	—		—	
No	1.11 (0.97-1.26)	0.13	1.15 (1.00-1.32)	0.04
**No Treatment**				
No	—		—	
Yes	0.98 (0.89-1.08)	0.70	0.88 (0.79-0.98)	0.02
**Class of Case**				
Dx & Treat	—		—	
Dx Only	1.91 (1.70-2.15)	<0.001	1.02 (0.88-1.19)	0.80
Non-Analytical	0.98 (0.87-1.10)	0.70	0.83 (0.72-0.97)	0.02
Treat Only	0.89 (0.84-0.95)	<0.001	1.24 (1.15-1.34)	<0.001
**Cancer Site**				
Digestive	—		—	
Breast	0.51 (0.47-0.56)	<0.001	0.19 (0.17-0.21)	<0.001
CLL[2] & Lymphoma	11.8 (10.4-13.2)	<0.001	1.49 (1.29-1.71)	<0.001
Male Genital	0.4 (0.37-0.44)	<0.001	0.06 (0.05-0.07)	<0.001
Other	4.25 (3.93-4.60)	<0.001	0.66 (0.60-0.72)	<0.001
Respiratory	1.49 (1.34-1.66)	<0.001	1.73 (1.57-1.89)	<0.001
Skin	0.59 (0.53-0.67)	<0.001	0.22 (0.19-0.25)	<0.001
Urinary	0.95 (0.86-1.05)	0.30	0.26 (0.23-0.29)	<0.001
**Insurance**				
Medicare	—		—	
Medicaid	0.91 (0.77-1.08)	0.30	1.53 (1.29-1.82)	<0.001
None	1.32 (1.07-1.62)	0.008	1.81 (1.45-2.25)	<0.001
Other	0.86 (0.44-1.69)	0.70	1.04 (0.48-2.28)	>0.90
Tricare	0.89 (0.75-1.06)	0.20	0.87 (0.71-1.07)	0.20
Unknown	0.94 (0.83-1.07)	0.40	1 (0.86-1.17)	>0.90

© 2024 Desjardins MR et al. *JAMA Network Open.*

Yes	0.96 (0.90-1.03)	0.30	0.9 (0.83-0.97)	0.008
**Zone * Race/Eth**				
>75%%-95% * Asian	0.79 (0.53-1.18)	0.20	0.75 (0.46-1.21)	0.20
>95% * Asian	1.5 (0.97-2.30)	0.06	1.64 (1.01-2.67)	0.04
>75%%-95% * Hispanic	0.77 (0.47-1.27)	0.30	1.01 (0.56-1.84)	>0.90
>95% * Hispanic	0.58 (0.32-1.05)	0.07	1.28 (0.67-2.44)	0.50
>75%-95% * Non-Hisp Black	1.19 (0.95-1.48)	0.13	0.88 (0.68-1.15)	0.40
>95% * Non-Hisp Black	1.02 (0.77-1.34)	>0.90	0.88 (0.63-1.22)	0.40
>75%-95% * Other	1.24 (0.72-2.13)	0.40	0.97 (0.49-1.93)	>0.90
>95% * Other	2.64 (1.41-4.96)	0.003	2.64 (1.22-5.72)	0.01
>75%-95% * Unknown	0.23 (0.08-0.61)	0.003	1.46 (0.51-4.18)	0.50
>95% * Unknown	0.97 (0.31-3.11)	>0.90	0.62 (0.07-5.84)	0.70
**CA Zone * CoC**				
>75%-95% * Dx Only	0.61 (0.42-0.89)	0.009	2 (1.35-2.95)	<0.001
>95% * Dx Only	0.49 (0.32-0.73)	<0.001	2.23 (1.46-3.42)	<0.001
>75%-95% * Non-Analytical	1.18 (0.96-1.45)	0.12	0.88 (0.67-1.16)	0.40
>95% * Non- Analytical	1.08 (0.86-1.37)	0.50	1.11 (0.82-1.50)	0.50
>75%-95% * Treat Only	0.91 (0.79-1.04)	0.20	1.31 (1.12-1.53)	<0.001
>95% * Treat Only	0.74 (0.63-0.87)	<0.001	1.08 (0.89-1.31)	0.40

[1]OR = Odds Ratio, CI = Confidence Interval
[2]Chronic lymphocytic leukemia

© 2024 Desjardins MR et al. *JAMA Network Open.*

eTable 5. Multinomial LR Results for 2015-2019 SKCCC Patient Cohort

**Stage at Diagnosis**	**Unknown Stage**		**Late-stage**	
**Variable**	**OR[1] (95% CI)**	**p-value**	**OR[1] (95% CI)**	**p-value**
**Zone**				
75% CA	—		—	
>75%-95%	0.99 (0.87-1.13)	0.90	0.72 (0.62-0.83)	<0.001
>95%	1.13 (0.93-1.36)	0.20	0.71 (0.57-0.88)	0.002
**Age**				
>75	—		—	
<22	3.3 (2.55-4.26)	<0.001	0.53 (0.36-0.76)	<0.001
22-45	1.12 (1.00-1.27)	0.05	0.78 (0.69-0.88)	<0.001
46-65	1.03 (0.94-1.14)	0.50	1.08 (0.99-1.18)	0.09
66-75	0.92 (0.85-1.00)	0.05	0.99 (0.92-1.07)	0.80
**Race/Ethnicity**				
Non-Hisp White	—		—	
Asian	0.79 (0.69-0.91)	0.001	0.96 (0.84-1.09)	0.50
Hispanic	1 (0.84-1.20)	>0.90	1 (0.84-1.19)	>0.90
Non-Hisp Black	1.26 (1.16-1.37)	<0.001	1.08 (1.01-1.17)	0.03
Other	1.18 (0.94-1.49)	0.20	1.07 (0.85-1.34)	0.60
Unknown	2.28 (1.59-3.28)	<0.001	0.83 (0.56-1.23)	0.30
**Female Sex**	0.71 (0.67-0.76)	<0.001	0.84 (0.78-0.89)	<0.001
**Tobacco Use**				
Yes	—		—	
No	1 (0.90-1.11)	>0.90	0.86 (0.79-0.93)	<0.001
**Alcohol Use**				
Yes	—		—	
No	1.01 (0.95-1.07)	0.80	0.97 (0.91-1.02)	0.20
**Surgery**				
Yes	—		—	
No	0.67 (0.61-0.75)	<0.001	3.17 (2.95-3.41)	<0.001
**Radiation**				
Yes	—		—	
No	1.19 (1.10-1.29)	<0.001	0.86 (0.80-0.91)	<0.001
**Chemo**				
Yes	—		—	
No	0.6 (0.54-0.66)	<0.001	0.29 (0.27-0.31)	<0.001
**Hormone Therapy**				
Yes	—		—	
No	1.61 (1.45-1.80)	<0.001	0.84 (0.77-0.91)	<0.001
**Immunotherapy**				
Yes	—		—	

© 2024 Desjardins MR et al. *JAMA Network Open.*

No	1.12 (0.99-1.26)	0.07	0.74 (0.67-0.81)	<0.001
**No Treatment**				
No	—		—	
Yes	3.08 (2.71-3.49)	<0.001	1.32 (1.19-1.45)	<0.001
**Class of Case**				
Dx & Treat	—		—	
Dx Only	1.01 (0.88-1.16)	0.90	1.06 (0.94-1.20)	0.30
Non-Analytical	0.48 (0.41-0.56)	<0.001	0.27 (0.23-0.32)	<0.001
Treat Only	0.87 (0.81-0.94)	<0.001	1.09 (1.02-1.17)	0.01
**Cancer Site**				
Digestive	—		—	
Breast	0.25 (0.22-0.29)	<0.001	0.23 (0.21-0.26)	<0.001
CLL[2] & Lymphoma	19.8 (17.4-22.4)	<0.001	1.38 (1.20-1.59)	<0.001
Male Genital	0.18 (0.16-0.21)	<0.001	0.5 (0.45-0.54)	<0.001
Other	6.35 (5.81-6.93)	<0.001	1 (0.92-1.09)	>0.90
Respiratory	0.62 (0.54-0.72)	<0.001	1.29 (1.17-1.41)	<0.001
Skin	0.63 (0.54-0.73)	<0.001	0.46 (0.39-0.53)	<0.001
Urinary	0.71 (0.63-0.80)	<0.001	0.48 (0.42-0.53)	<0.001
**Insurance**				
Medicare	—		—	
Medicaid	0.95 (0.78-1.15)	0.60	1.54 (1.29-1.84)	<0.001
None	1.48 (1.08-2.04)	0.01	2.46 (1.84-3.27)	<0.001
Other	1.24 (0.53-2.92)	0.60	0.81 (0.30-2.17)	0.70
Tricare	0.84 (0.70-1.01)	0.07	0.75 (0.63-0.90)	0.002
Unknown	2.11 (1.44-3.09)	<0.001	1.49 (1.0-2.24)	0.05
Yes	0.94 (0.87-1.02)	0.20	0.94 (0.88-1.01)	0.11
**Zone * Race/Eth**				
>75%-95% * Asian	1.09 (0.71-1.68)	0.70	0.76 (0.49-1.17)	0.20
>95% * Asian	1.57 (0.90-2.73)	0.11	2.01 (1.19-3.41)	0.009
>75%-95% * Hispanic	1.35 (0.78-2.32)	0.30	0.66 (0.35-1.23)	0.20
>95% * Hispanic	0.89 (0.48-1.67)	0.70	0.68 (0.35-1.30)	0.20
>75%-95% * Non-Hisp Black	1.06 (0.81-1.37)	0.70	1.03 (0.81-1.31)	0.80
>95% * Non-Hisp Black	0.82 (0.55-1.23)	0.30	1.28 (0.90-1.81)	0.20
>75%-95% * Other	1.29 (0.70-2.36)	0.40	0.67 (0.33-1.34)	0.30
>95% * Other	1.17 (0.55-2.45)	0.70	0.96 (0.43-2.16)	>0.90
>75%-95% * Unknown	0.7 (0.25-1.98)	0.50	1.78 (0.49-6.44)	0.40
>95% * Unknown	1.4 (0.37-5.36)	0.60	1.57 (0.38-6.44)	0.50
**Zone * CoC**				
>75%-95% * Dx Only	1.22 (0.82-1.84)	0.30	0.96 (0.68-1.36)	0.80
>95% * Dx Only	0.94 (0.53-1.64)	0.80	0.92 (0.57-1.49)	0.70
>75%-95% * Non-Analytical	0.82 (0.63-1.08)	0.20	0.79 (0.56-1.11)	0.20

© 2024 Desjardins MR et al. *JAMA Network Open.*

>95% * Non- Analytical	0.94 (0.67-1.31)	0.70	0.68 (0.44-1.05)	0.08
>75%-95% * Treat Only	0.97 (0.82-1.14)	0.70	1.64 (1.39-1.94)	<0.001
>95% * Treat Only	0.99 (0.79-1.24)	>0.90	1.33 (1.06-1.69)	0.01

[1]OR = Odds Ratio, CI = Confidence Interval

[2]Chronic lymphocytic leukemia

© 2024 Desjardins MR et al. *JAMA Network Open.*
