# Supplementary material for: Disparities in Cancer Stage Outcomes by Catchment Areas for a Comprehensive Cancer Center
Source: JAMA Netw Open. 2024 May 2;7(5):e249474. doi: 10.1001/jamanetworkopen.2024.9474 (PMC11066700; doi:10.1001/jamanetworkopen.2024.9474)
Supplement: Supplement 2. — Data Sharing Statement [file jamanetwopen-e249474-s002.pdf]

Data Sharing Statement

Desjardins. Disparities in Cancer Stage Outcomes by Catchment Areas for a Comprehensive Cancer Center. *JAMA Netw Open*. Published May 02, 2024.
doi:10.1001/jamanetworkopen.2024.9474

Data
Data available: No

Additional Information
Explanation for why data not available: Census data is publicly available, while the cancer data is restricted due to data-use agreements.
